# Cation Exchange and Spontaneous Crystal Repair Resulting in Ultrathin, Planar CdS Nanosheets

**DOI:** 10.1021/acs.chemmater.3c01900

**Published:** 2023-09-28

**Authors:** Maaike M. van der Sluijs, Jara F. Vliem, Jur W. de Wit, Jeppe J. Rietveld, Johannes D. Meeldijk, Daniel A. M. Vanmaekelbergh

**Affiliations:** † Condensed Matter & Interfaces, Debye Institute for Nanomaterials Science, Utrecht University, 3584 CC Utrecht, The Netherlands; ‡ Electron Microscopy Centre, Utrecht University, Universiteitsweg 99, 3584 CG Utrecht, Netherlands

## Abstract

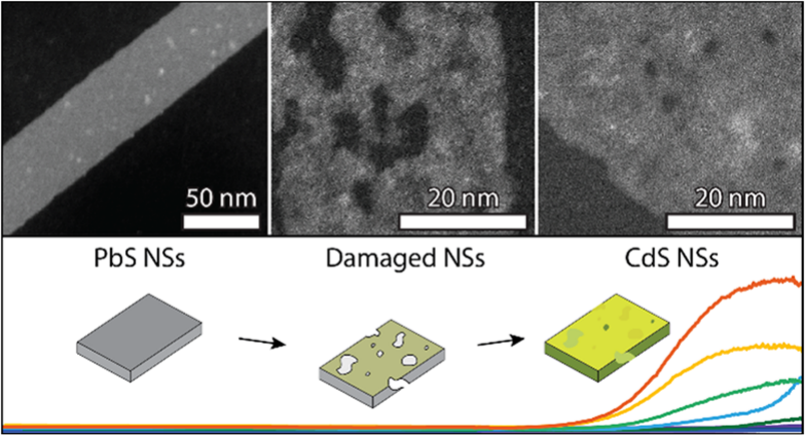

Cation exchange has become a major postsynthetic tool to obtain nanocrystals with a combination of stoichiometry, size, and shape that is challenging to achieve by direct wet-chemical synthesis. Here, we report on the transformation of highly anisotropic, ultrathin, and planar PbS nanosheets into CdS nanosheets of the same dimensions. We monitor the evolution of the Cd-for-Pb exchange by *ex-situ* TEM, HAADF-STEM, and EDX. We observe that in the early stages of the exchange the sheets show large in-sheet voids that repair spontaneously upon further exchange and annealing, resulting in ultrathin, planar, and crystalline CdS nanosheets. After cation exchange, the nanosheets show broad sub-band gap luminescence, as often observed in CdS nanocrystals. The photoluminescence excitation spectrum reveals the heavy- and light-hole exciton features, with very strong quantum confinement and large electron–hole Coulomb energy, typical for 2D ultrathin Cd-chalcogenide nanosheets.

## Introduction

Extensive research over the last two decades has developed cation exchange into a powerful postsynthetic tool to obtain colloidal quantum materials that are unattainable, or challenging to achieve by direct synthesis pathways. Colloidal nanocrystals (NCs) enable ion-exchange protocols with reasonable temperature and time scales (minutes to hours). The NCs feature abundant facet/solvent interfaces, and only nanometer-scale diffusion paths need to be overcome to accomplish ion replacement. Specifically, cation exchange has been used to convert NCs with an AX stoichiometry (A being the cation, X being the anion) into BX NCs. It is a process that can be used to completely exchange NCs,^1−3^ or to form heterostructures (AX/BX) with atomically sharp interfaces and band gap offsets.^4−6^ From a crystallographic viewpoint, there are two modes of exchange. In the first mode, the new cations spread homogeneously through the crystal replacing the original cation; a type of exchange which has for instance been used for impurity doping with magnetic cations.^7−9^ In the second mode, a BX phase replaces the AX phase and heterointerfaces form when the cation exchange is arrested before completion,^1,10,11^ when the facet reactivity toward exchange varies over the structure,^12,13^ or when the AX and BX phases have immiscible crystal structures.^14−17^ To study the exchange in detail, NCs with a strong anisotropic shape are beneficial as they offer opportunities for TEM monitoring of the process in a specific orientation, e.g. crystallographic domains that proceed in specific directions and atomic reconfigurations during or after the exchange process.^10,18,19^ For instance, in 2D CdSe nanoplatelets, the Pb-for-Cd exchange proceeds via PbSe crystal growth, starting from the edges of the nanoplatelets. The interface extends further into the interior upon prolonged exchange, retaining the 2D nature of the nanoplateletss while the corners and lateral dimensions lose definition.^3,10^

Recently, there have been significant advances in the synthesis and study of ultrathin, highly anisotropic PbS nanosheets (NSs) with a deformed orthorhombic crystal structure. Despite having a thickness of only a few nanometers and lateral dimensions in the range of 100–200 nm, the PbS NSs exhibit a rectangular and planar shape, appearing completely flat in TEM without visible distortion, or curling.^20,21^ This makes them compelling candidates to study the crystallographic aspects of cation exchange. Moreover, strain within CdS NSs was previously shown to strongly perturb the band structure and induce band gap variations up to 20 meV,^22^ in addition to influencing the self-assembly of the NSs.^23,24^ Thus, distortion in NSs can hamper potential applications and device design. The cation exchange process might offer pathways to ultrathin CdS NSs with extended lateral dimensions and no curling, unlike NSs prepared by direct synthesis.^25−28^

Here, we report on a study of Cd^2+^-for-Pb^2+^ cation exchange in highly anisotropic ultrathin PbS nanosheets (NSs). We followed the incorporation of Cd^2+^ ions into the PbS NSs with *ex-situ* TEM and EDX mapping, which shows the homogeneous distribution of the Cd^2+^ over the NSs from the early stages of exchange. It is an indication that the Cd^2+^-for-Pb^2+^ exchange occurs randomly via the top and bottom facets of the NSs with multiple nucleation points of the CdS. In the early stages of the ion exchange, the sheets show significant voids. Remarkably, this damage becomes less prominent in the ensemble upon extended annealing and exchange. Both intact and damaged CdS NSs are observed in a 1:1.5 ratio and we discuss the potential self-repair mechanism in detail. The exchanged planar CdS NSs have lateral dimensions similar to those of the parent PbS NSs, albeit with a slight increase in thickness (0.5–1 nm).

While the original narrow band gap PbS NSs show no luminescence,^20,21^ the exchanged NSs begin to show a broad luminescence peak centered around 700 nm when roughly 80% of the cations are exchanged. This large redshift with respect to the wide bulk band gap energy (2.42 eV^29^) indicates that trap-emission is the dominant recombination mechanism. Probing the broad emission band with excitation spectroscopy, the heavy-hole and light-hole transitions are observed at 3.1 and 3.35 eV, while the onset of free carrier absorption is at 3.8 eV. Comparing these results to the experimental results for CdS nanoplatelets,^25^ the strong confinement energy and large electron–hole Coulomb interaction (heavy-hole at 3.1 eV versus free carrier absorption at 3.8 eV = 0.7 eV) agree with the ultrathin dimensions of 2.2 ± 0.4 nm of the CdS sheets and the low dielectric constant of the crystal and the environment.^30^

## Experimental Section

### Chemicals

Acetonitrile (ACN, anhydrous, 99.8%), 1-butanol (BuOH, 99.8%), cadmium acetate dihydrate (Cd(Ac)_2_·3H_2_O), lead(II) thiocyanate (Pb(SCN)_2_ 99.5%), methanol (MeOH, 99.8%), 1-octadecene (ODE, 90%), oleic acid (OA, 90%), oleylamine (OLAM, 99%), selenium powder (Se mesh, 99.99%), and tetrachloroethylene (TCE) were purchased from Sigma-Aldrich. Hexane (anhydrous, 99%) and toluene (anhydrous, 99.8%) was purchased from Alfa Aesar.

### Lead Sulfide Nanosheet Synthesis

Lead sulfide (PbS) nanosheets (NSs) were prepared following a previously reported synthesis procedure by Akkerman et al.^20^ In a typical synthesis, 32.3 mg (0.1 mmol) of Pb(SCN)_2_ was added to a 25 mL three-neck flask with 223.8 mg (0.250 mL) of OA, 101.6 mg (0.125 mL) of OLAM, and 7.9 g (10 mL) of ODE. To dissolve the Pb(SCN)_2_ the flask was capped with a Vigreux, two septa and heated in air to 110 °C for 30 min (oxygen is necessary for the decomposition). Then, the temperature was quickly increased to 165 °C at a rate of ∼15 °C per minute. Around 155 °C the transparent reaction mixture turns light brown, and when the temperature reaches 165 °C the flask is quickly cooled with a water bath. The NSs were washed by centrifugation at 2750 rpm (840 RCF), the clear supernatant was removed, and the precipitate was redispersed in 5 mL of toluene. As an additional washing step, 5 mL of acetonitrile was added before centrifuging again at 2750 rpm. The supernatant was removed and the precipitate was redispersed in in 5 mL of toluene.^21^ See Figures S1 and S2 for the characterization of PbS NSs.

### Cation Exchange of the Lead Sulfide Nanosheets to Cadmium Sulfide Nanosheets

A 0.35 M solution of Cd(OA)_2_ was prepared following a procedure by Casavola et al.,^1^ which results in an excess of OA remaining in the precursor. From the PbS NSs dispersion, 2 mL was dried in vacuum, and the sheets were redispersed in 5 mL of ODE by sonication. In a N_2_ filled glovebox, an aluminum block with a reference vial containing 7 mL ODE and a thermometer was heated to the desired reaction temperature (usually 100 °C). When the temperature of the block had stabilized, a stirring bar and 2 mL of a 0.35 M Cd(OA)_2_ solution were added to the prepared PbS NSs. The experiment time starts when the vial is placed in the preheated block, where the PbS NSs were kept for the duration of the reaction (1, 2.5, 10, 15, 30, 60, 120, or 240 min) and then allowed to cool to room temperature. At longer reaction times, the initially dark brown dispersion changes color to light brown or even orange (Figure S3). It is important to note that it takes about 3 min until the dispersion reaches 90 °C, and another 10 min to reach 100 °C (Figure S4 shows a temperature trace).

In addition to the standard cation exchange procedure discussed above, more experiments were performed with varied reaction parameters. An important additional experiment consisted of adding 0.25 mL of 0.35 M Cd(OA)_2_ after 2 h at 100 °C, followed by a temperature increase to 130 °C for 1 h. Then, another 0.25 mL of Cd(OA)_2_ was added before another temperature increase to 150 °C for the final hour. The resulting dispersion has a distinct yellow color (Figure S3). When discussed, the results from this cation exchange procedure will be referred to as “240 min high temperature” (240 min high T). It is important to note that byproducts occasionally formed in the PbS NSs synthesis are rock salt PbS cubes (∼16.7 nm) and thicker 2D “bones”. As these byproducts undergo the cation exchange procedure, they retain the majority of their Pb center while surrounded by completely exchanged NSs (Figure S17).

When the dispersion had cooled to room temperature, the vial was centrifuged at 2750 rpm (840 RCF) for 10 min. The clear supernatant was removed by pipet, and the precipitate was redispersed in 1 mL toluene. Then, 1 mL of methanol and 2 mL of butanol were added as antisolvent, and the dispersion was again centrifuged at 2750 rpm for 10 min. The precipitate was redispersed in 1 mL of toluene. This washing step was repeated when needed.

### Characterization

TEM samples were prepared by dropcasting a dilute dispersion of NCs on carbon-coated copper Formvar TEM grids. To induce stacking of the sheets, MeOH/BuOH (1:2) was added to the dispersion before dropcasting. An edge-up, stacked orientation of the sheets was achieved, which allows for imaging in the lateral direction of the PbS nanosheets. To reduce hydrocarbon contamination during imaging, the TEM sample was treated with EtOH and activated carbon following a previously published procedure.^31^ BF-TEM imaging was performed on a Thermo Fisher Talos L120C, or on a Fei Talos F200X operating at respectively 120 and 200 keV. Low-resolution HAADF-STEM images were acquired on a Fei Talos F200X operating at 200 keV. Atomically resolved high-resolution HAADF-STEM imaging was performed on a double aberration-corrected Thermo Fisher Spectra 300, operating at an accelerating voltage of 300 keV.

Ultraviolet-visible (UV/vis) absorption spectra were recorded on a PerkinElmer 950 UV/vis/NIR spectrophotometer with quartz cuvettes. The samples for these measurements were prepared by drying the NSs in vacuum, after which they were redispersed in 2 mL hexane. Photoluminescence measurements were performed on an Edinburgh Instruments FLS920 spectrometer equipped with TMS300 monochromators and a 450 W Xe lamp. To all samples dispersed in 1 mL of toluene, we added an additional 0.5 mL of toluene before transferring the sample to a quartz cuvette. With an excitation wavelength of 390 nm, the measurements were performed from 420 to 800 nm in steps of 2 nm with a dwell time of 1 s, an excitation slit width of 10 nm, and an emission slit width of 5 nm. A 416 nm long-pass filter was placed in the emission path. Photoluminescence excitation measurements were performed with the same setup without the filter by monitoring the emission at 650 nm upon excitation from 260 to 500 nm. A step size of 2 nm, dwell time of 1 s, and 10 nm slit width were used. The recorded emission spectra were corrected for the spectral responsivity of the detectors and monochromators.

Proton nuclear magnetic resonance (^1^H NMR) measurements were performed using an Agilent MRF400 instrument equipped with a OneNMR probe and Optima Tune system. Spectra were recorded according to the following parameters: 400 MHz, CDCl_3_ 25 °C. In short, 0.6 mL of the CdS NSs dispersion was dried and redispersed in 0.6 mL CDCl_3_. The samples were then measured with a long relaxation delay (25 s) to allow complete relaxation.^32,33^

## Results

### Original PbS Nanosheets and CdS Nanosheets after Cation Exchange

**Figure 1:**
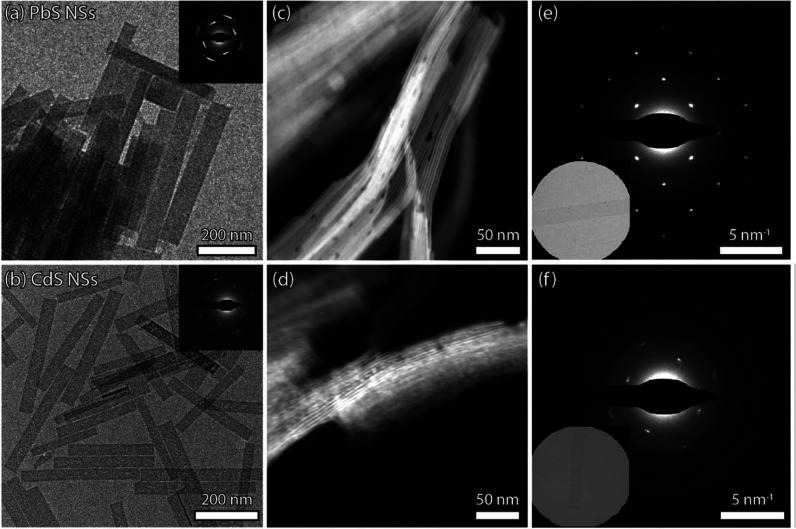
Overview TEM images of the original PbS NSs (a) and CdS NSs after 120 min of cation exchange (b); the insets show the corresponding SAED patterns. The sharp corners and lateral dimensions of the parent PbS NSs are preserved in the exchange. In parts (c) and (d), the stacked NSs show a slight increase in thickness to 2.2 ± 0.4 nm (Figure S2). The SAED of the overview (a and b) and the single NSs (e and f) indicate a change in crystal structure from deformed orthorhombic PbS to a CdS crystal structure with broadened diffraction maxima (see Supporting Information Section S1 for further discussion).

To study the Cd^2+^-for-Pb^2+^ cation exchange in 2D materials, we selected PbS NSs as synthesized by Akkerman et al.^20,21^ as the parent NSs (Figure 1a). We then followed a procedure by Casavola et al., developed for cation exchange in anisotropic PbSe/CdSe core/shell nanomaterials.^1^ To PbS NSs dispersed in ODE, a cadmium oleate solution (Cd(OA)_2_) is added before heating the vial to temperatures in the range of 100–150 °C with reaction times up to 4 h (see the Experimental Section). After 120 min at 100 °C, the PbS NSs with a deformed orthorhombic crystal structure have undergone a complete cation exchange to CdS NSs. The large lateral dimensions and sharp 90° corners of the PbS NSs (Figure 1a,b) are generally preserved in this reaction. There is a slight reduction of the average length from 231 to 205 nm, accompanied by a small decrease in width from 37 to 30 nm (Figure S1). Moreover, despite the pronounced structural anisotropy, no visible curling or folding of the thin sheets is observed (different from directly synthesized CdS and CdSe nanoplatelets).^25,26^ By examining vertical stacks of the NSs in HAADF-STEM mode, we determined the thickness of the respective PbS and CdS sheets (Figure 1c,d). The parent PbS sheets were previously shown to be either 1.2 or 1.8 nm thick;^21^ while the CdS sheets are slightly thicker at 2.2 ± 0.4 nm (Figure S2a). As indicated by the selected area electron diffraction (SAED) of a single nanosheet (inset) in Figure 1e,f, the sharp diffraction maxima of the deformed orthorhombic crystal structure have broadened and shifted, possibly due to misalignments in the crystal structure. However, characterization of the crystal structure in the CdS NSs has been challenging due to the limited number of reflections, thin material, and similarity in potential crystal structures (see Supporting Information Section S1 for further discussion).

### Evolution of the Cd^2+^-for-Pb^2+^ Ion Exchange Process

**Figure 2:**
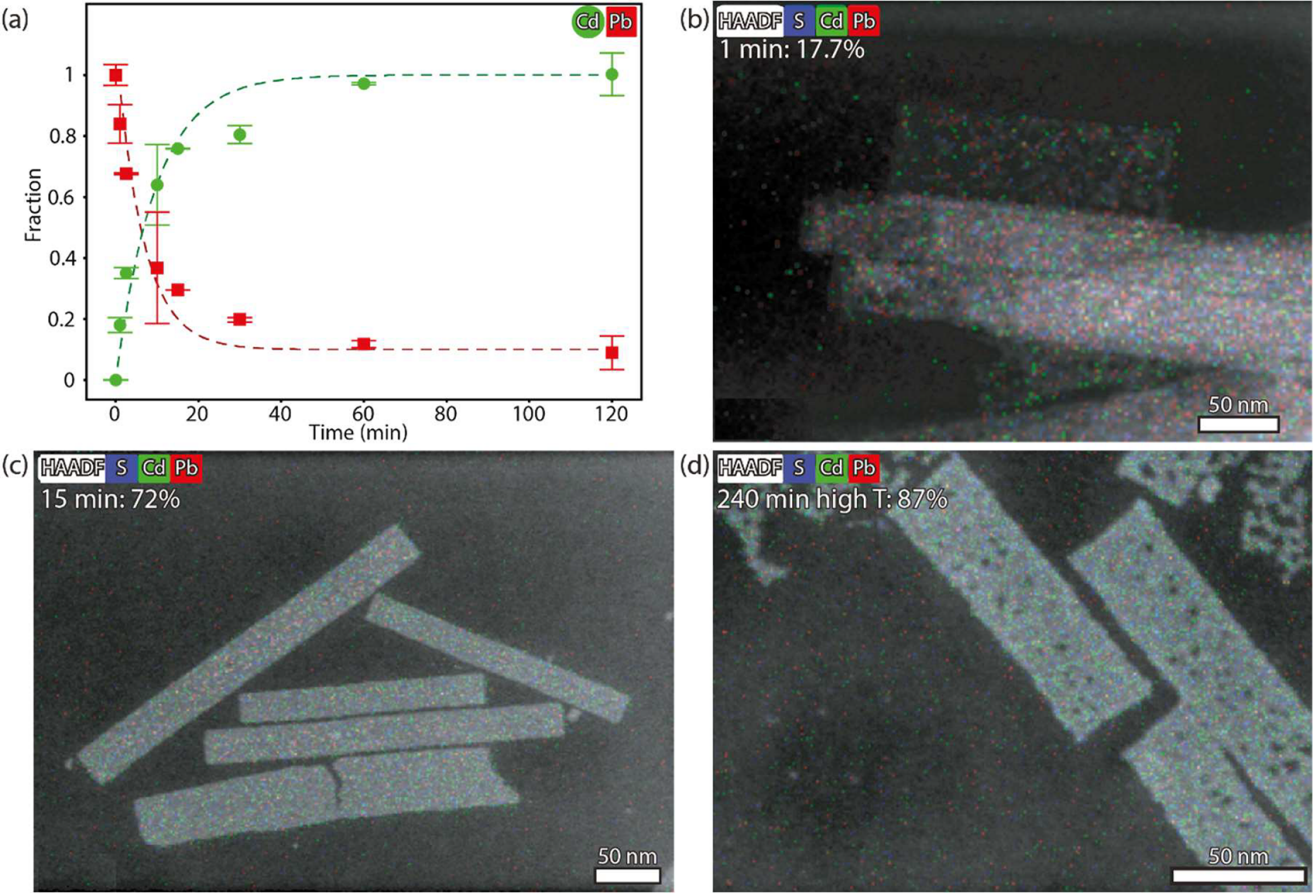
Cation exchange in the NSs was studied ex-situ with ensemble EDX and STEM-EDX measurements. (a) Plot of the respective Pb and Cd fractions (normalized with respect to the percentage of Pb in the original PbS NSs) as a function of the reaction time. Initially the parent PbS NSs contain 54% S and 46% Pb. After 120 min at 100 °C, a complete Cd^2+^-for-Pb^2+^ is achieved with 46% Cd (green) and 4% residual Pb (red). The dark green line is a fit to the cadmium fraction with the first Langmuir isotherm (θ(*t*) = 1 – *e*^–*kt*^). In all EDX maps (b–d), the Cd is homogeneously distributed in the NSs. In Figure S10 additional maps and quantification are shown.

To monitor the cation exchange over time, experiments were performed at 100 °C for up to 120 min, sometimes with a prolonged reaction using additional Cd(OA)_2_ and a higher temperature (up to 150 °C, see Experimental Section: sample 240 min high T). All intermediate reaction products were studied ex-situ by HAADF-STEM and energy dispersive X-ray spectroscopy (EDX) to follow the cation exchange progression. With ensemble EDX measurements, the Cd fraction of the total cation percentage was determined. On a linear time scale, this fraction initially increases quickly, before saturating around 60 min (Figure 2a, Table S5 and Figure S9). The dark green line is a fit of the Cd fractions with the first Langmuir isotherm and will be discussed below. Within one min of placing the reaction vial with Cd(OA)_2_ and the parent PbS NSs (46% Pb and 54% S) in a preheated aluminum block (100 °C) the NSs contain Cd (a fraction of 0.18, green). Concurrently, the fraction of Pb decreases (with 0.16, red), indicating that the exchange begins well before the dispersion reaches the reaction temperature of 100 °C (∼3 min to reach 90 °C, Figure S4). In Supporting Information Table S6 we discuss some additional experiments that show the reaction will even start at room temperature, although higher temperatures are necessary for it to proceed. After 10 min, more than half of the cations in the sheets are Cd (a fraction of 0.65). Then, the exchange rate decreases, and complete replacement is achieved after 120 min (a fraction of 1, 46% Cd) while some residual Pb remains (fraction of 0.1, ∼5 ± 2%). During the entire exchange, the S content stays between 55 and 50% (Figure S9).

With STEM-EDX mapping, we studied the distribution of the elements in the NSs (Figure 2b–d, with additional maps and quantification in Figure S10). Already after one min both Pb and Cd (red and green) are homogeneously distributed (Figure 2b). This distribution is then maintained throughout the reaction (15 min in Figure 2c), and upon complete exchange a small fraction of Pb remains. However, the residual Pb is not concentrated around the NSs and instead randomly distributed in the maps (both after 120 min at 100 °C, Figure S10f, and with a prolonged reaction at higher temperature, Figure 2d). When quantifying a single sheet (indicated by the white box in Figure S10), these maps show the same trend as the ensemble EDX measurements previously discussed (Figure 2a, Table S5).

The persistent homogeneous distribution of both cations in the sheets indicates that the Cd^2+^-for-Pb^2+^ cation exchange does not proceed from the edges, as previously observed for 2D zinc blende CdSe nanoplatelets. There, a PbSe deposit forms at the edge of the nanoplatelet due to the strong passivation of the top and bottom facets with carboxylic acid.^27,34^ Over time these initial deposits extend into the host and the resulting nanoplatelets have a distorted lateral shape with an increased polydispersity in thickness.^3,10,35^ In the cation exchange of PbS NSs studied here, the homogeneous distribution of the elements occurs in the zone axis. Pb-for-Cd replacements can take place in two different ways: by a genuine CdS/PbS crystal interface proceeding across the NSs or alternatively by starting at individual and randomly dispersed atomic crystal positions. STEM analysis of vertically stacked sheets could be very helpful, as this would enable the study of an alternate zone axis. However, the resolution of the elemental maps of NSs in this orientation was too low due to instability in the NSs during exchange, hydrocarbon contamination (despite applied cleaning techniques^31^) and image drift (Figure S11).

Fortunately, the plot of the ensemble EDX measurements provides some insight. The increase of the cadmium fraction (θ) follows the first Langmuir function (dark green line in Figure 2a which is discussed in Supporting Information Section S3). This points to random exchanges on an atomic level, as a linear increase of the Cd fraction would be expected in case of a progressing CdS/PbS reaction front. Thus, this suggests that the rate-limiting step in the exchange is the random adsorption of the cations to appropriate surface sites, not the ion diffusion into the structure. This is reasonable, especially considering the fact that the parent PbS NSs are only 4 or 6 monolayers thick (1.2 to 1.8 nm) and thus more than 50% (4 MLs) or 33% (6 MLs) of the cations are at the surface and accessible.

### Self-Repair of CdS Nanosheets

**Figure 3:**
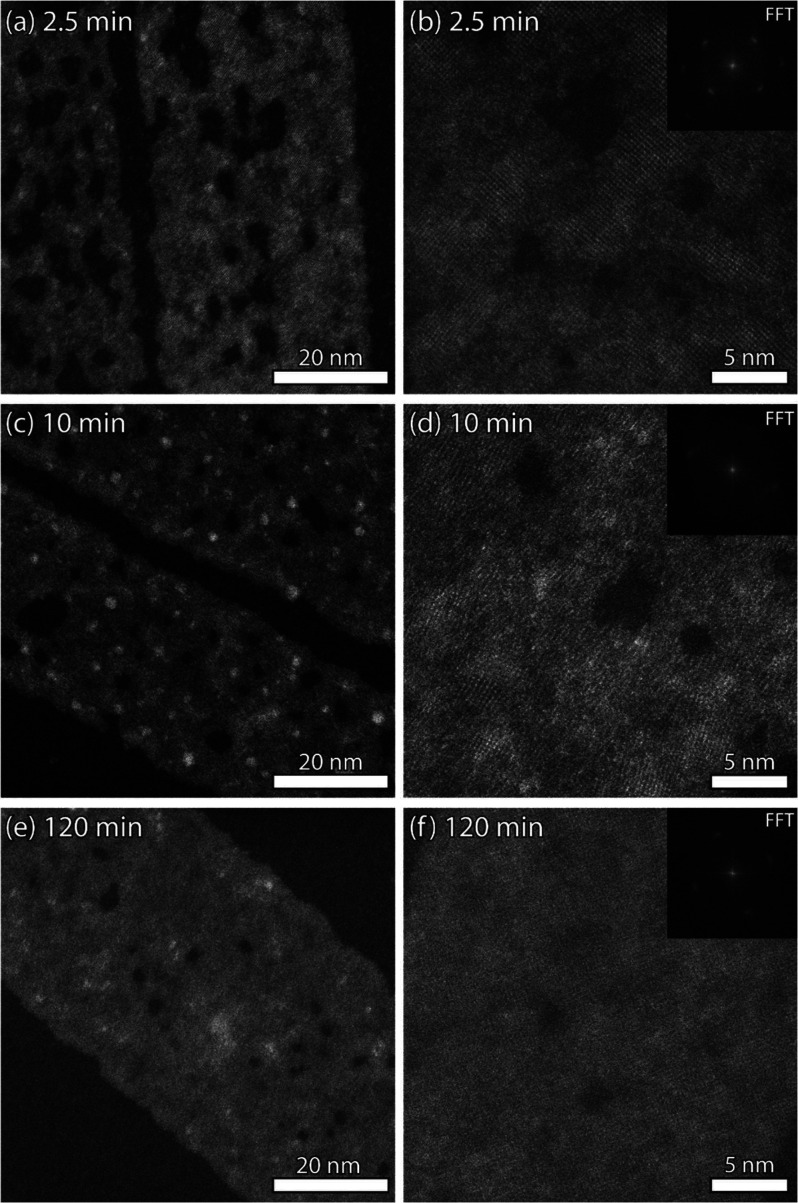
Structure and crystallinity of NSs during cation exchange. In (a) and (b) the NSs are shown with atomic resolution after 2.5 min of exchange. The sheets have large voids, connected by amorphous areas, that are still present after 10 min (c and d). After 120 min the Pb is completely exchanged, the voids have almost disappeared and the overall crystallinity of the NSs has improved (e and f).

A detailed study of the intermediary products with HAADF-STEM shows that although the CdS NSs retain the rectangular shape of the parent NSs, the process of cation exchange causes many voids in the structure. Such “damaged” sheets are particularly abundant in the early stages of the reaction (Figure 3a). The voids in the sheets are surrounded by both amorphous and crystalline areas (Figure 3b), but a corresponding Fourier transform (inset) shows the uniform orientation of the crystalline domains with only a slight broadening of the diffraction maxima. This retention of orientation is reminiscent of the formation of the parent PbS NSs, where early in the synthesis crystallographic domains form with remarkable alignment of orientation in rectangular but amorphous or pseudocrystalline self-induced templates.^21^ In the early stages of the cation exchange the number of damaged sheets is high (>80%, n = 18 sheets after 1 and 2.5 min of reaction, see Figure 3a, Figure S14). At 10 min more than >95% of the sheets are damaged (n = 48). However, as the exchange reaction continues, the quality of the sheets improves; the number and size of the voids in the sheets decrease, while the crystalline area increases (Figure 3c and 3e). At the end of the exchange, 40% of the sheets are intact. Still, 60% of the NSs are damaged, although the degree of damage per NS varies greatly (Figure S13).

The decrease of the voids and increase in crystallinity with time reflect a remarkable self-repair of the NSs. Evidently, the ultrathin sheets experience harsh conditions in the exchange reaction. While we observe a comparative exchange of cations, a local imbalance in stabilizing ligands or cations can cause the fast deterioration of the thin sheets. The subsequent improvement in the quality of the CdS NSs indicates that a repair takes place. The CdS units necessary for the repair may originate from the same sheet (a genuine self-repair), resulting in sheets with smaller lateral dimensions. Alternatively, the required CdS units may originate from strongly damaged NSs that are sacrificed for the repair of others. This is a reasonable assumption since the percentage of damaged sheets is high. With this “parasitic repair by Ostwald ripening”, the thermodynamically more stable NSs are repaired at the expense of those that are less stable and damaged. Of course these two mechanisms could occur concomitantly, and additional research will be necessary to understand the exact process occurring here.

### Optical Properties of the CdS Nanosheets

In absorption spectra, two-dimensional CdS nanoplatelets obtained by direct wet-chemical synthesis have shown heavy-hole (HH) and light-hole (LH) excitonic transitions. The energy of these transitions depends strongly on the thickness of the platelets (bulk CdS has a band gap of 2.42 eV). In zinc blende CdS nanoplatelets, the absorption features are observed around 3.8 eV (320 nm) when only ∼1.2 nm thick, while they are around 2.8 eV (440 nm) when ∼2 nm thick. Typically, the thinnest nanoplatelets have a very weak emission peak from their HH exciton-to-ground-state transition while there is no luminescence detected from the thickest nanoplatelets (∼2 nm).^25^ In addition to the band gap emission, some sub-band gap photoluminescence is observed, commonly referred to as trap emission. These charge-carrier traps are usually attributed to structural imperfections or uncoordinated (surface) atoms and a lot of research has focused on the prevention and repair of these imperfections in NCs.^36−39^ However, the direct study of these traps has been challenging on both an ensemble level and for individual NCs due to the heterogeneity of traps and the wide range of time scales involved in trap-related dynamics.^40,41^

**Figure 4:**
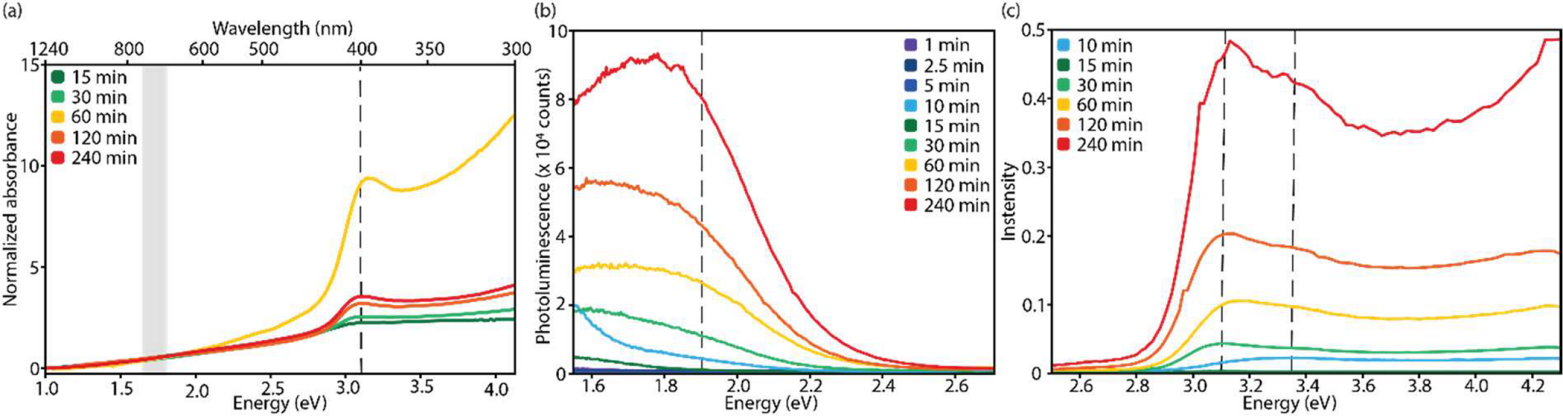
Absorption, photoluminescence, and photoluminescence excitation spectroscopy measurements were recorded for CdS NSs exchanged for 1 to 240 min at 100 °C. (a) Absorption measurements of the NSs when dispersed in hexane. Each sample displays considerable scattering at long wavelengths. For clarity, the measurements were normalized at 1.55 eV (800 nm). All CdS NSs have a transition around 3.1 eV (400 nm, indicated by the dashed line), which is a large shift when compared to the PbS sheets (1.8 eV, indicated by the gray box). (b) Photoluminescence spectra of all intermediate cation exchange samples excited at 390 nm. Initially no luminescence is observed, but after 10 min, a broad subgap emerges below 2.2 eV which increases over time. (c) The photoluminescence excitation spectra as monitored at 1.9 eV (650 nm), indicated by the dashed line in (b). The heavy-hole and light-hole exciton peaks are observed at 3.1 and 3.35 eV, respectively (indicated by the black dashed lines), while free carrier absorption starts at 3.8 eV.

In a NS suspension, scattering by the large CdS sheets makes it difficult to extract quantitative information from the absorption spectra (Figure 4a). However, a clear transition is observed around 3.1 eV (400 nm) in all measurements. This is a large blueshift compared to the parent PbS NSs which showed a transition between 1.6 and 1.8 eV (indicated by the gray box).^21^ Unlike the parent PbS sheets, the exchanged CdS sheets show a broad sub-band gap luminescence transition when excited at 390 nm (Figure 4b). A low background of trap-emission emerges below 2 eV after 10 min of exchange (over 60% of the cations are Cd), when still more than 80% of the NSs are heavily damaged (Figure 3c,d). Furthermore, from 30 min onward there is an increase in trap luminescence with increasing reaction time. In the final CdS NSs (after 120–240 min at 100 °C) a broad luminescence feature is observed between 2.4 and 1.6 eV. Band-gap luminescence would be expected below 2.8 eV but is not observed in any measurement, even with an additional 120 min of annealing at 100 °C or a prolonged cation exchange at higher temperature. On the other hand, the intensity of the trap-luminescence does increase significantly (Figure S16).

To gain additional insight into the optical transitions of the CdS sheets, we performed photoluminescence excitation spectroscopy for intermediate CdS/PbS structures and the final CdS NSs (Figure 4c). While monitoring the luminescence at 1.9 eV (650 nm), the spectra gradually show the (HH, CB) and (LH, CB) excitonic transitions and the onset of free carrier generation, typical for a 2D quantum well.^25^ The typical heavy-hole and light-hole exciton peaks emerge at 3.1 and 3.35 eV respectively, and the absorption increases again after 3.8 eV. These excitonic transitions are at higher energy than those of the directly synthesized CdS platelets,^25^ reflecting very strong confinement in the thickness direction. Moreover, the electron–hole binding energy is as high as 0.7 eV, due to the thinness of the sheets and low dielectric constant. These ultrathin CdS NSs (2.2 ± 0.4 nm) thus show a strong quantum confinement and a large electron–hole binding energy. Based on the optical measurements, we conclude that our CdS NSs have strong excitonic and band-to-band absorption, enhanced by a very high exciton binding energy. Emission spectroscopy shows that this results in only broad trap-emission with a large Stokes shift. To obtain excitonic photoluminescence, both structural improvement after cation exchange and improvement of the surface passivation are needed.

In summary, we have obtained colloidal CdS NSs by Cd^2+^-for-Pb^2+^ exchange in PbS NSs, with the latter having a deformed orthorhombic structure. These CdS NSs have an average thickness of 2.2 ± 0.4 nm and lateral dimensions of 205 nm by 30 nm with remarkably straight edges. The exchange process was monitored with ex-situ analysis of the intermediate reaction products. The Cd^2+^-for-Pb^2+^ exchange initially progresses exponentially until saturation is achieved, similar to the case for the first Langmuir absorption isotherm. We interpreted this as the random adsorption of Cd^2+^ cations at appropriate surface sites being the rate-limiting step in the exchange, not the diffusion of cations into the ultrathin structure. In addition, EDX maps showed the continued homogeneous distribution of the Cd^2+^ ions, already from the earliest stages of the exchange. Thus, the exchange occurs via the large top and bottom facets of the NSs.

The NSs show significant damage from the early stages of cation exchange; both amorphous regions and large voids are observed within the still rectangular sheets. Upon extended cation exchange and continued annealing, these voids become less prominent in the ensemble, indicating that self-repair takes place in up to 40% of the NSs. The final CdS NSs remain planar, retaining the highly anisotropic shape with lateral dimensions similar to those of the parent PbS NSs.

While the original narrow band gap PbS NSs show no luminescence, the NSs begin to show excitonic absorption features typical for ultrathin CdS NSs after 10 min of exchange. They begin to show broad, weak luminescence centered at around 700 nm. The large redshift with respect to the band gap indicates that trap emission is the dominant radiative recombination mechanism. Upon probing the broad emission band with photoluminescence excitation spectroscopy, features of the heavy-hole and light-hole transitions are observed at 3.1 and 3.35 eV respectively, while the onset of free carrier absorption is at 3.8 eV. In conclusion, this study highlights the successful synthesis of colloidal, ultrathin, but planar CdS NSs via cation exchange. After which a self-repair mechanism results in more crystalline sheets showing trap luminescence.

## Supporting Information Available

Histogram showing the lateral dimensions of PbS NSs and CdS NSs after cation exchange; HAADF-STEM images of stacked CdS NSs and corresponding histograms; picture showing the color change of the dispersion during cation exchange; plot of the temperature during exchange; ^1^H NMR spectra of exchanged CdS NSs; table detailing the resonances in the NMR spectra; Section S1 detailing the crystal structure study of the CdS NSs; Section S2 detailing the compositional analysis of the CdS NSs with EDX; additional HAADF-STEM images and EDX maps of the NSs during cation exchange; HAADF-STEM images of stacked CdS NSs and corresponding EDX maps; Section S3 detailing a model for adsorption of cations to a surface; HAADF-STEM images and histogram showing the quality of the NSs before and after exchange; histogram showing the quality of the NSs during cation exchange; table detailing the experimental parameters of additional exchange experiments; histogram showing the quality of the NSs in the additional experiments; photoluminescence emission an excitation measurements of exchanged CdS NSs; HAADF-STEM images and EDX maps of side-products formed in PbS NSs syntheses after cation exchange; and photoluminescence measurements showing a Raman scattering feature in early cation exchange samples (PDF)

## Abbreviations

2D: two-dimensional
AX/BX: A or B cations with X being the anion
(BF-)TEM: bright-field transmission electron microscopy
CB: conduction band
CdS: cadmium sulfide
HAADF-STEM: high-angle annular dark-field scanning transmission electron microscopy
HH: heavy-hole exciton features
LH: light-hole exciton feature
MLs: monolayers
NCs: nanocrystals
NMR: nuclear magnetic resonance
NSs: nanosheets
PbS: lead sulfide
RPM: revolutions per minute
SAED: selected area electron diffraction
UV/vis: ultraviolet–visible spectroscopy
XRD: X-ray diffraction
