# Supplementary material for: Cation Exchange and Spontaneous Crystal Repair Resulting in Ultrathin, Planar CdS Nanosheets
Source: Chem Mater. 2023 Sep 28;35(19):8301–8. doi: 10.1021/acs.chemmater.3c01900 (PMC10568967; doi:10.1021/acs.chemmater.3c01900)
Supplement: Supplementary file 1 — cm3c01900_si_001.pdf [file cm3c01900_si_001.pdf]

Cation Exchange and Spontaneous Crystal Repair Resulting in Ultrathin, Planar CdS Nanosheets - Supporting Information

Maaike M. van der Sluijs[1], Jara F. Vliem[1], Jur W. de Wit[1], Jeppe J. Rietveld[1], Johannes D. Meeldijk[2], Daniel A. M. Vanmaekelbergh[1*]

1 Condensed Matter & Interfaces, Debye Institute for Nanomaterials Science, Utrecht University, 3584 CC Utrecht, The Netherlands.

2 Electron Microscopy Centre, Utrecht University, Universiteitsweg 99, 3584 CG Utrecht, Netherlands

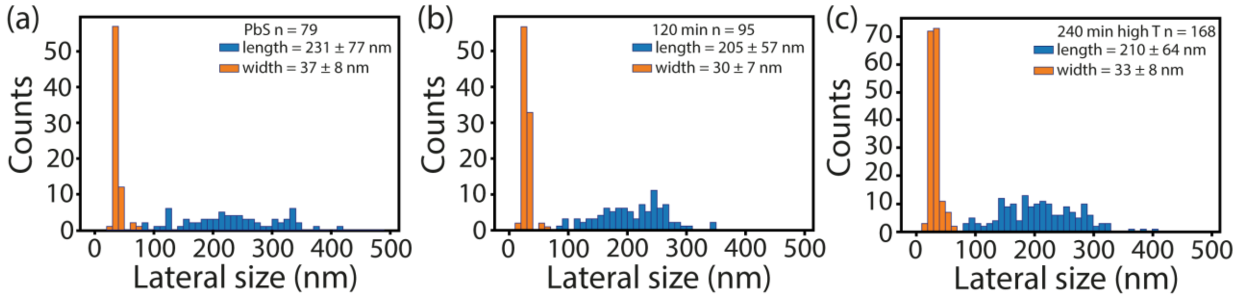

Figure S1. The lateral dimensions of the NSs were determined from HAADF-STEM images for the parent PbS NSs (a) and after undergoing cation exchange (b and c). The histograms have a bin size of 10 nm. (a) The parent PbS NSs have an average length of 231 ± 77 nm and width of 37 ± 8 nm. As these PbS NSs undergo cation exchange for 120 minutes at 100°C (b), or even for a prolonged reaction at higher temperature (c and see Experimental Section) the average length decreases to 205 ± 57 and 210 ± 64 nm. This decrease is predominantly due to a decline in the number of NSs longer than 300 nm. In addition, a slight decrease in width is observed as well, although still within the reported NS dimensions.

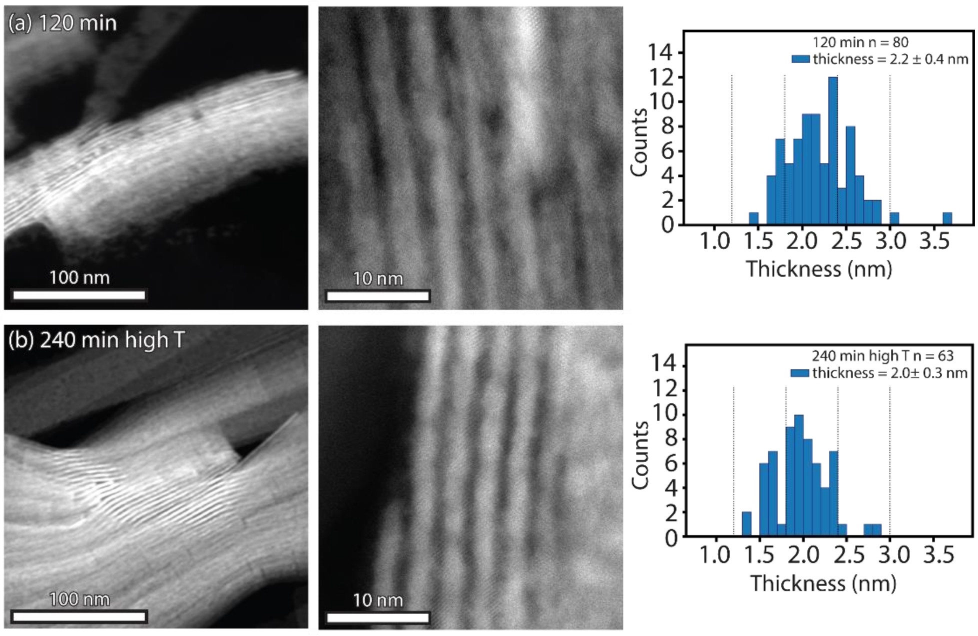

Figure S2. The thickness of the parent PbS NSs was previously reported to be a 1:1 population of 1.2 and 1.8 nm (4 or 6 orthorhombic monolayers respectively).[1] After cation exchange, both with the 120 minutes and the 240 min high temperature procedure, the average thickness has increased towards 2.2 ± 0.4 nm (a) and 2.0 ± 0.3 nm (in b). In TEM images it is clearly observed that both thicker and thinner areas are present; for characterization we measured the thicker areas of the NSs.

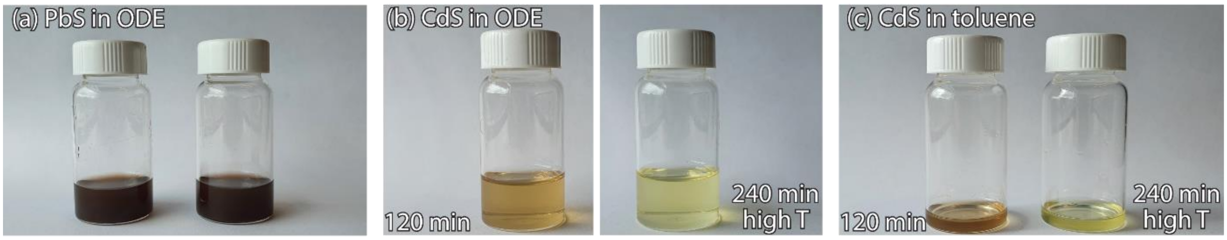

Figure S3. Pictures of the PbS NSs before (a) and after cation exchange (b and c). The initially dark brown dispersion turns light brown or orange, or even yellow in case of prolonged reaction times at higher temperatures. After purification, these colours are retained (c).

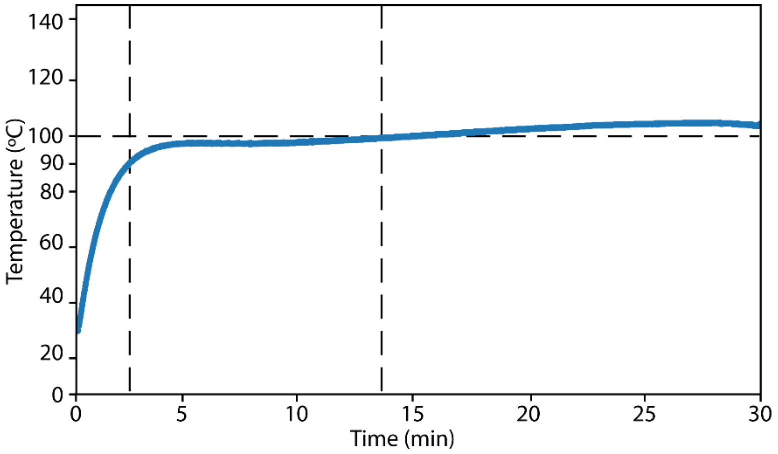

Figure S4. The temperature trace of a vial containing 7 mL ODE upon placement in a preheated (100°C) aluminium block at time 0. It takes about 3 minutes for the solution to reach 90°C, but another 10 minutes to fully reach 100°C.

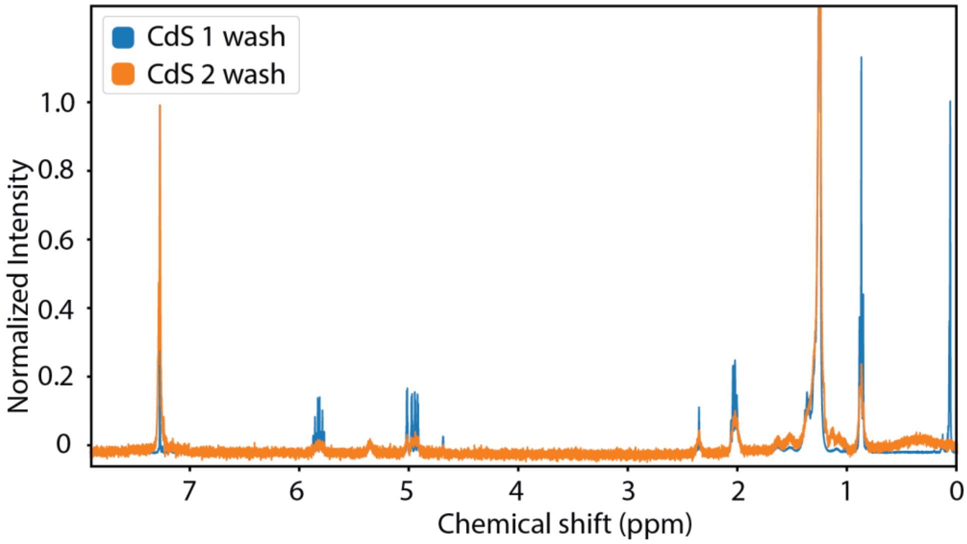

Figure S5. ^{1}H-NMR spectra of exchanged CdS NSs (120 min) after 1 and 2 rounds of purification (Experimental Section). **Table** S1 gives an overview of each peak. ODE is still present in the dispersion after 1 washing step (the sharp resonances at 5.8 and 4.9 ppm). The small 5.3 ppm resonance is attributed to the presence of OA. The relatively sharp peak does not show broadening due to binding to the surface of the NSs, as was previously observed both in 3D and 2D materials.[2-4] After the second washing step both the OA and ODE features decrease.

Table S1. List of the resonances observed in **Figure** S5 and attributed to the protons present in the CdS dispersion. Note that thiocyanate, the ligand on the parent PbS NSs does not contain any protons and therefore cannot be characterized with ^{1}H-NMR.

**Chemical shift (ppm)**	**Corresponds to**	**Group**
0.9	OA, ODE	**- CH_3**
1.3	OA, ODE	**-CH_2**
2.0	OA, ODE	**-CH_2, -NH_2**
2.3	OA	**O=C—CH_3**
4.9	ODE	**=CH_2**
5.3	OA	**-HC=CH-**
5.8	ODE	**-CH=**CH_2
7.3	$CDCl_3$	-

Section S1. Crystal structure of the CdS NSs

Table S2. A list of potential crystal structures of the CdS NSs.

Crystal structure	Compound	ICSD #	a (Å)	b (Å)	c (Å)
Deformed orthorhombic	PbS	14945	11.9	4.22	4.22
Orthorhombic	CdS	600773	3.471	4.873	3.399
Rock salt	CdS	52825	5.3	5.3	5.3
Wurzite	CdS	154186	4.1365	4.1365	6.7160
Zinc blende	CdS	81925	5.830	5.830	5.830

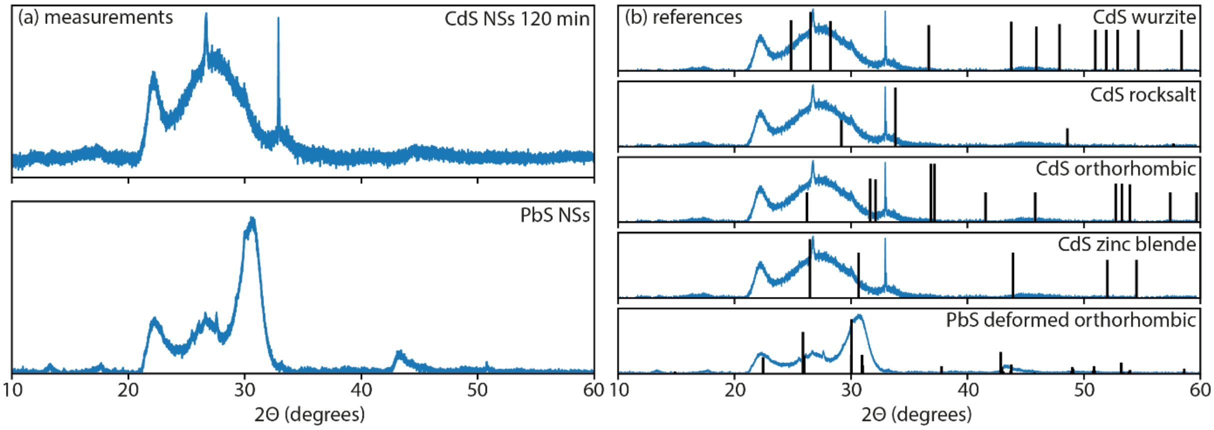

Figure S6. (a) For XRD measurements, the PbS NSs (bottom) and CdS NSs (top) dispersions were dropcasted on a silicon wafer. The parent PbS NSs pattern has broad peaks between 20° and 35°, and the CdS NSs pattern has very similar peaks that are further broadened and shifted to slightly higher degrees. (b) When comparing the XRD measurements to reference data of the deformed orthorhombic crystal structure (ICSD: 14945), the peak position of the PbS NSs fits quite well. However, the relative intensity of the experimental peaks does not match, which could be due to some preferred orientation of the NSs. We compare the CdS NSs to potential crystal structure candidates: being zinc blende (ICSD: 81925), orthorhombic (ICSD: 600773), rock salt (ICSD: 528255) and wurzite (ICSD: 154186), but no pattern fits completely. Although wurzite and zinc blende result in better fits than the other structures. Specifically, the reflection at 22° only corresponds exclusively to the deformed orthorhombic PbS crystal structure. A possible explanation could be residual PbS, but the intensity is high when compared to EDX measurements (**Table** S5 residual Pb of only ~5%).

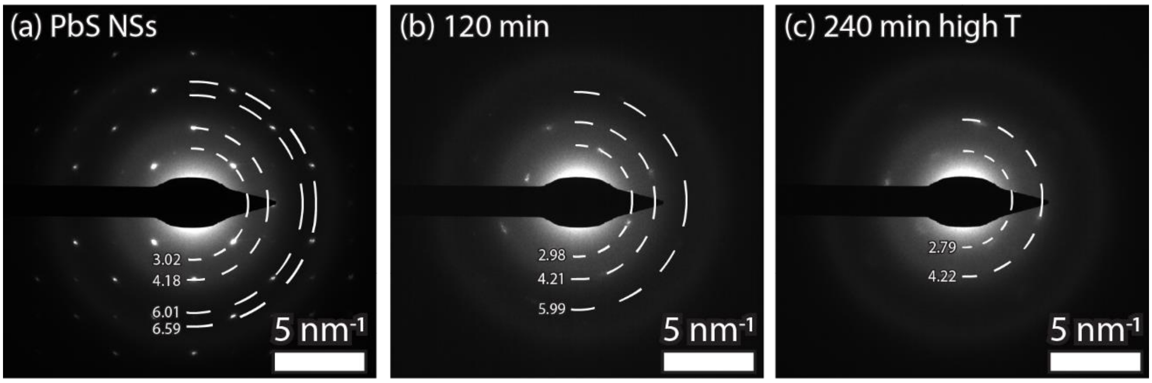

Figure S7. The SAED patterns of the single NSs in **Figure** 1 were azimuthally integrated with CrystBox software.[5] In all diffractograms each ring has been labelled with the peak position, 2π/d in nm^{-1}. The interplanar distances corresponding to these values are listed in **Table** S3.

Table S3. The interplanar distances corresponding to the peak positions in **Figure** S7.

**PbS (Å)**	**CdS 120 min (Å)**	**CdS 240 min high T (Å)**
3.31	3.35	3.59
2.39	2.38	2.37
1.66	1.67	-
1.52	-	-

Due to the 2D ultrathin nature of the NSs studied here, many of the reflections ordinarily observed in XRD and SAED are lacking. As we only observe 2 or 3 rings clearly in SAED it has been challenging to assign a crystal structure to the CdS NSs. In **Table** S4 we have indicated potential lattice spacings in bold (within 0.07 Å) for each potential crystal structure of the CdS NSs (b and c in **Figure** S7). The deformed orthorhombic crystal structure of the parent PbS NSs overlaps with the 2.38 and 1.67 lattice spacings but is lacking the 3.35. All other candidates have lattice spacings similar to the CdS NSs, although the orthorhombic and wurzite structure have the most overlap. We therefore cannot definitively assign a crystal structure to the CdS NSs. In view of the fact that the CdS sheets are very thin, and the parent PbS sheets have a deformed orthorhombic crystal structure it is not unthinkable that the strain of the ligands induces deformation from a conventional structure.

Table S4. The d-spacings of the potential crystal structures of the NSs observed in **Figure** 1, **Figure** S7. We have selected the potential lattice spacings observed in the CdS samples from **Table** S3 within 0.07, to compensate for the manual calibration of the Crystbox software.

CdS NSs SAED (Å)	Deformed orthorhombic (Å)	Orthorhombic (Å)	Wurzite (Å)	Zinc blende (Å)	Rock salt (Å)
**3.35**	3.96	**3.40 (001)**	**3.58 (100)**	**3.37 (111)**	3.06
**2.38**	3.44	2.82	**3.36 (002)**	2.92	2.65
**1.67**	2.97	2.79	3.16	2.06	1.87
-	2.88	2.44	**2.45 (012)**	1.76	**1.6 (311)**
-	2.66	**2.43 (101)**	2.06	**1.68 (222)**	-
**-**	**2.43 (410)**	2.17	1.98	-	-
**-**	**2.38 (311)**	1.74	1.90	-	-
-	2.11	**1.72 (121)**	1.79	-	-
**-**	**1.65 (611)**	**1.70 (002)**	1.76	-	-
**-**	**1.57 (701)**	1.60	1.73	-	-
-	-	1.55	**1.68 (004)**	-	-
-	-	-	1.58	-	-

Section S2. Compositional analysis with EDX

The compositional analysis of the cation exchange at different stages has been performed by Energy dispersive X-ray Spectroscopy (EDX) analysis. EDX spectra were acquired for samples deposited on a TEM grid prepared as discussed in the Experimental Section. **Figure** S8 shows the representative spectra of PbS NSs and exchanged CdS NSs (240 min high T).

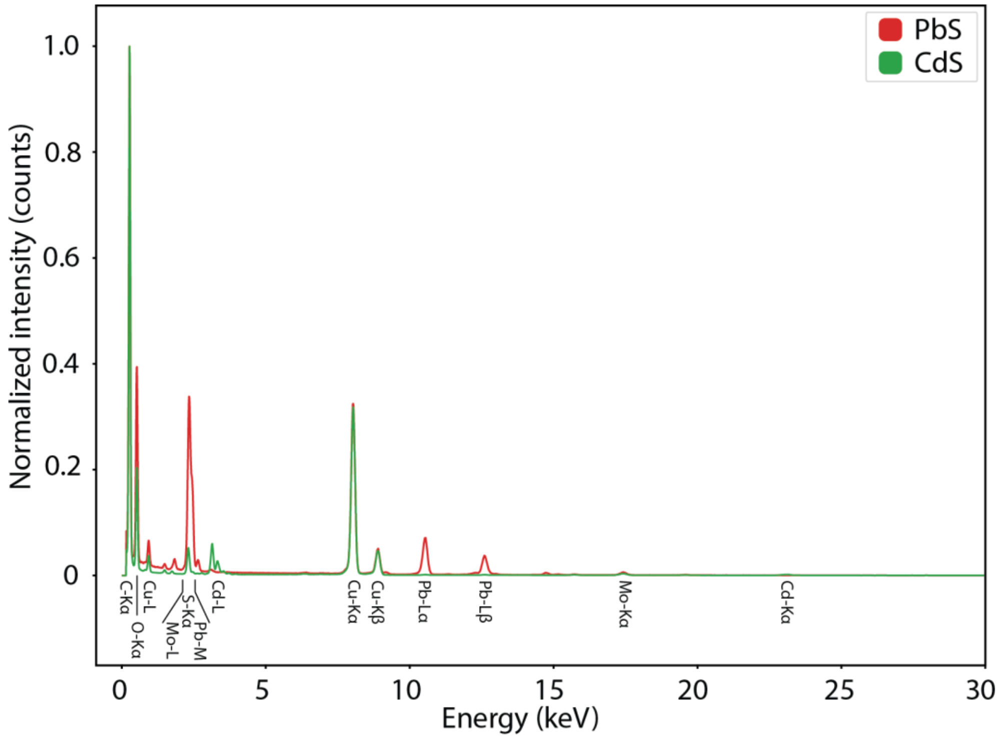

Figure S8. Representative bulk EDX measurements of the parent PbS NSs and exchanged CdS NSs on copper formvar-carbon TEM grids. To quantify the elements the S-Kα lines, the Pb-Lα lines and Cd-Lα lines were used, as these have limited overlap with other elements. In some measurements, a small amount of molybdenum was detected, which is possibly due to some parts of the microscope.

In **Table** S5 we report the quantification of the bulk EDX measurements. Each individual measurement has an uncertainty of ± 5%, but in the table we report the average percentage of each element and the standard deviation based on measuring at least 3 different areas on the TEM grids (except for the 15 minutes sample). In the last column of the table we report the percentage of cation exchange (% CE), determined as the percentage of cadmium over the total fraction of cations in the sheets at that stage.

Table S5. The average bulk EDX results for all experiments performed at 100°C.

Bulk EDX measurements 100°C	% Cd	% Pb	% S	% CE (% Cd of cations)
**0 min (n=3)**	0	46 ± 1.57	54 ± 1.57	0
**1 min (n=3)**	8.29 ± 1.13	38.63 ± 2.9	53.1 ± 1.87	17.7
**2.5 min (n=3)**	16.12 ± 0.83	31.12 ± 0.15	52.76 ± 0.95	35.1
**10 min (n=6)**	29.43 ± 6.07	16.92 ± 8.4	53.65 ± 1.28	63.5
**15 min (n=1)**	34.9	13.6	51.5	72
**30 min (n=3)**	37 ± 1.35	9.16 ± 0.13	53.8 ± 1.37	80.2
**60 min (n=3)**	44.7 ± 0.2	5.43 ± 0.54	49.77 ± 0.47	89.2
**120 min (n=6)**	46.1 ± 3.2	4.1 ± 2.53	50.12 ± 0.84	91.8
**-**	-	-	-	-
**240 min high T (n=3)**	42.03 ± 0.49	6.09 ±0.16	51.83 ± 0.4	87.3

Table S6. The average bulk EDX results for additional low temperature cation exchange experiments. At lower temperatures (room temperature or 50°C for 120 minutes) the cation exchange proceeds at a slower rate and does not result in complete exchange.

Bulk EDX measurements	% Cd	% Pb	% S	% CE
**Room temperature 120 min (n=3)**	5.55 ± 0.38	38.23 ± 0.86	56.23 ± 0.55	12.7
**50 °C 120 min (n=3)**	7.18 ± 0.14	35.1 ± 0.62	57.7 ± 0.56	17

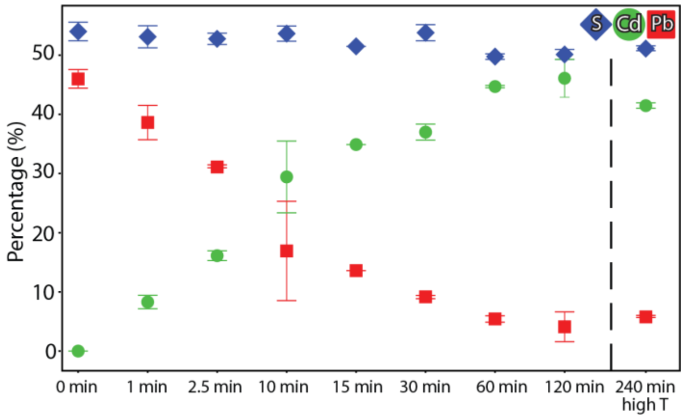

Figure S9. The percentages of sulfur, lead and cadmium as reported in **Table** S5 are plotted over time. The PbS NSs initially contain 54% sulfur and 46% lead and after 120 minutes at 100 °C a complete Cd^{2+}-for-Pb^{2+} is achieved. After 60 minutes of cation exchange a small decrease in sulfur is observed (from ~55% to 50%).

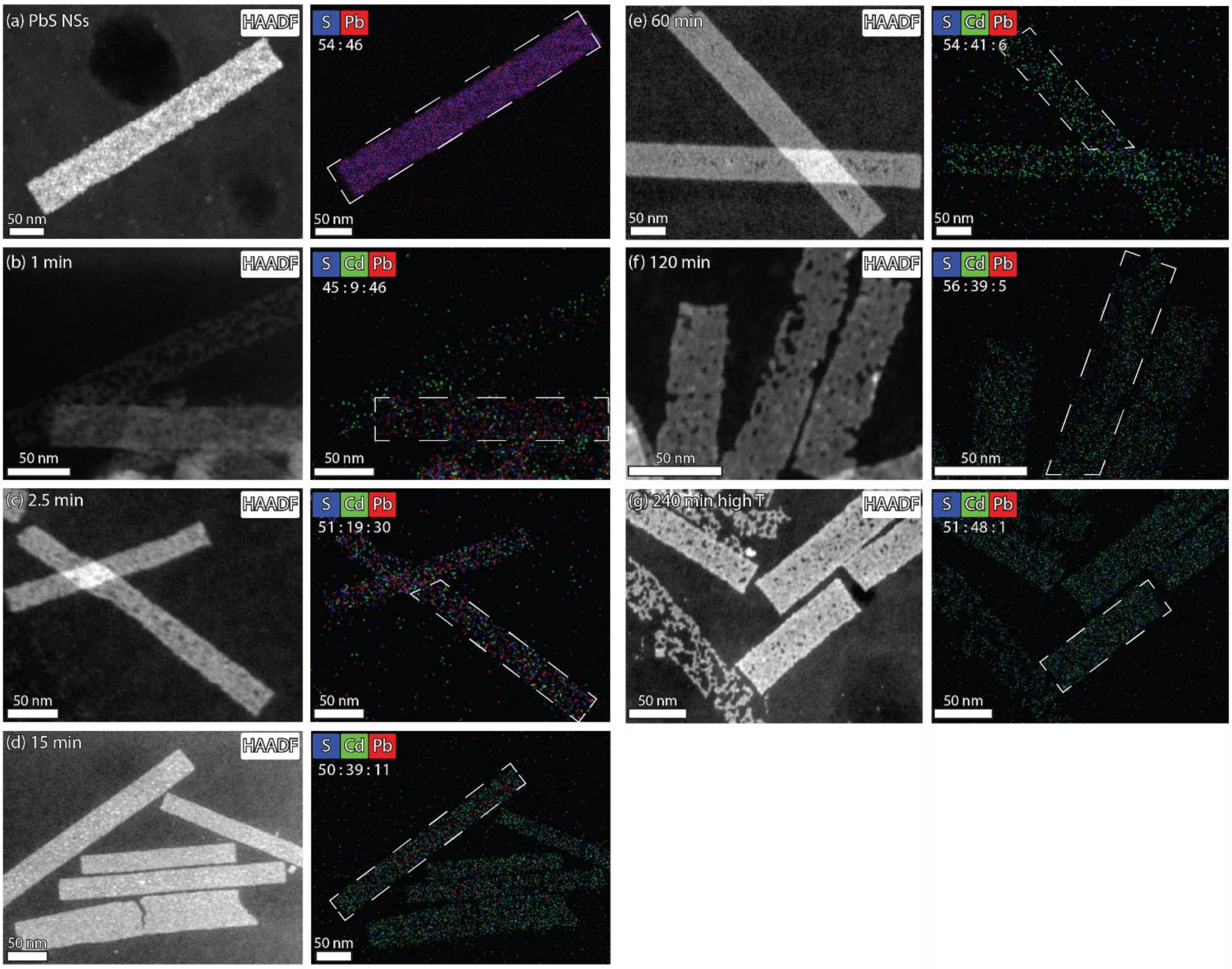

Figure S10. HAADF-STEM images (left) and their corresponding EDX maps (right). The single NSs as indicated by the white boxes are quantified and the elemental contents are reported in the top left of each map. These single NSs follow the same trend as the bulk EDX measurements reported in **Table** S5. Note that this technique provides information about single (or a few) NSs, but does not contain ensemble information. During the cation exchange, each map (b-g) shows a homogeneous distribution of cadmium and lead within the sheets. Late in the exchange (f-g), the remaining lead ions are primarily present in the background of the maps. We observe both intact and damaged NSs in the HAADF-STEM images, we will discuss this further below.

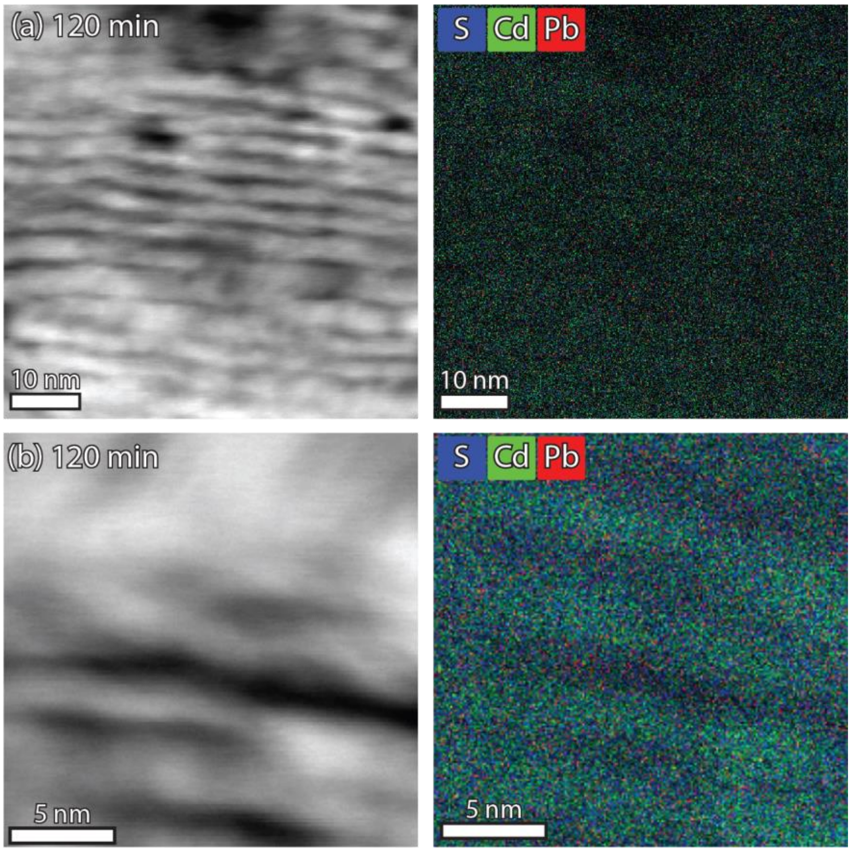

Figure S11. HAADF-STEM images of stacked CdS NSs at a 10 nm (a) and a 5 nm scale (b). The corresponding EDX maps were measured until hydrocarbon contamination resulted in image drift. They have a low signal, making the stacked sheets difficult to discern, but we tentatively conclude that the residual lead is randomly distributed in the maps.

Section S3. Model for adsorption to a surface

When modelling the kinetic process of reactions, a conventional approach is to simplify the calculations to the slowest step, as that rate is the bottleneck for the overall rate.[6] This simplification allows for the description of heterogeneous processes by well-known equations, originally developed for homogeneous systems. The PbS NSs are very thin, consisting of only 4 or 6 monolayers (1.2 to 1.8 nm). Thus, the number of readily available surface atoms in a NS is more than 50% (4 MLs) or more than 33% (6 MLs). The entire surface of the NSs is readily accessible when they are dispersed in a solvent, meaning that only two or four additional monolayers need to be traversed by diffusion. Therefore, we consider the rate-limiting step in the cation exchange to be the adsorption of the cadmium ions onto specific surface sites of the sheets. Labelling the fraction of sites on the surface available for Cd^{2+} adsorption as θ(t), the random adsorption on these sites can be described by:

$$\frac{d\theta}{dt} = k(1-\theta)$$

Resulting in:

$$\theta(t) = 1 - e^{-kt}$$

At 0% exchange, all the potential sites on the surface are still free (θ = 0), while at 100% exchange (46% cadmium in the sheets) every potential site is occupied by a Cd^{2+} adsorbate (θ = 1). It is a molecular adsorption process at a limited number of atomic positions. In **Figure** S12 we plot the fractions of cadmium and lead on a linear time scale, and fit the datapoints with the above equation. The exchange follows this trend quite well, with an initial high reaction rate that decreases after 15 minutes and saturates around 1 hour.

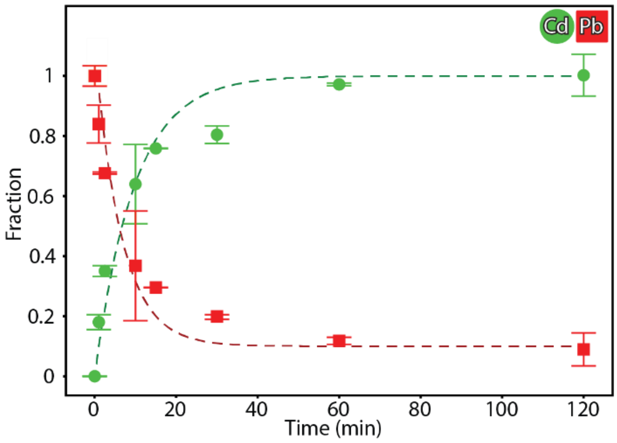

Figure S12. The fractions of cations as determined with bulk EDX measurements are plotted on a linear timescale. As the lead atoms in the PbS NSs are replaced with cadmium, the original fraction of 1 (46%) fully reduces to 0.09 (4.1%). The dark green and dark red dashed lines show the fit of the datapoints. The cadmium trend was fit with the first Langmuir isotherm: $\theta(t) = 1 - e^{-kt}$ and the lead trend with its reverse: $\theta(t) = e^{-kt} + 0.09$ (with 0.09 being the residual lead in the sample).

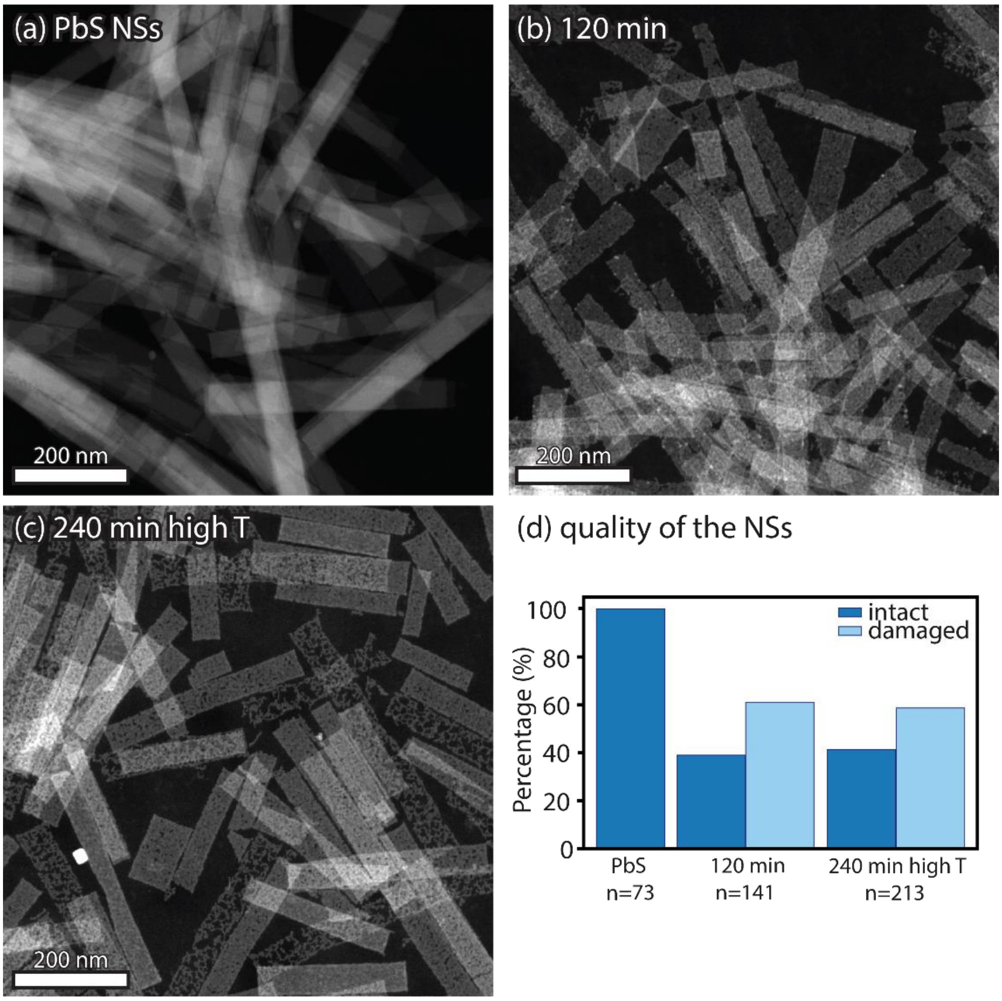

Figure S13. The parent PbS NSs were recently shown to display some small scale defects,[1] but during the cation exchange part of the CdS NSs are noticably damaged. We deem the parent PbS NSs to be intact (a), while after 120 minutes of cation exchange ~60% of the remarkably rectangular NSs has large holes (b). After additional time at higher temperature (240 min high T), the damaged NSs still comprise ~60% of the population (c). (d) A histogram showing the percentages of the intact and damaged sheets.

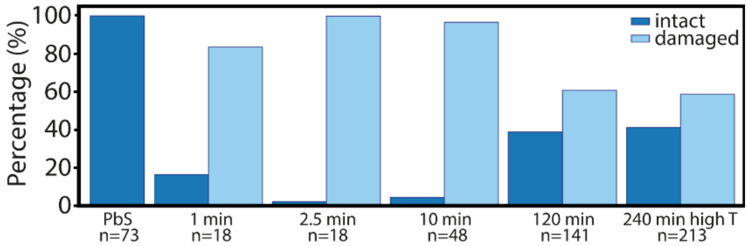

Figure S14. To study the quality of the NSs throughout the cation exchange we characterized the CdS NSs with HAADF-STEM. They are characterized as either intact or damaged i.e. containing large holes within the rectangular sheet. Due to clustering the 1 and 2.5 minute samples only a few NSs could be characterized, but the majority is damaged (>80%). In the 10 minute sample a larger population could be characterized and more than 90% of the NSs are damaged. An additional 110 minutes of cation exchange brings the percentage of damaged NSs down to 61% with no significant improvement in the 240 minutes high temperature experiment (59%). Thus, the initial PbS NSs quickly deteriorate resulting in damage in the majority of the NSs, although later in the cation exchange these holes appear to repair resulting in a lower degree of damage.

Table S7. The experimental parameters of the samples characterised in **Figure** S15.

Experiment	Initial $Cd(OA)_2$	100 °C	Additional reactant	130 °C	150 °C
**CdS 120 min**	2 mL 0.35 M	2 hours	-	-	-
**CdS 240 min high T**	2 mL 0.35 M	2 hours	2x 0.25 mL 0.35 M	1 hour	1 hour
**CdS 240 min high T with OA**	2 mL 0.35 M	2 hours	2x 0.25 mL OA	1 hour	1 hour
**CdS 4 mL 0.1 M**	4 mL 0.1 M	2 hours	-	1 hour	1 hour
**CdS 4 mL 0.35 M**	4 mL 0.35 M	2 hours	-	1 hour	1 hour

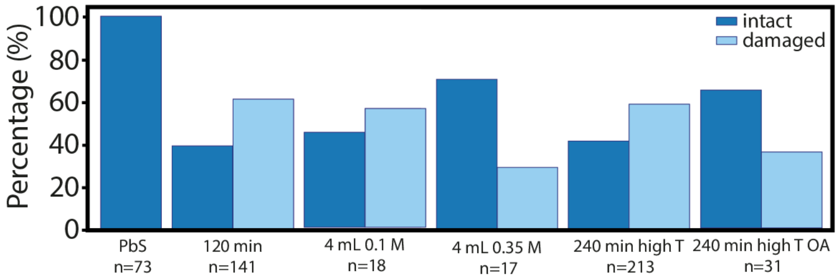

Figure S15. The CdS NSs of the additional experiments described in **Table** S7 were characterized with HAADF-STEM. Here, we doubled the amount of cadmium precursur added and we varied the concentration of the precursor. The 4 mL 0.1 M precursor improved the number of intact NSs slightly when compared to the 120 min sample (~55%), but 4 mL 0.35 M precursor improved the yield of intact NSs significantly to ~70%. Further experiments at a higher temperature with additional OA appear to also improve the quality of the NSs (~65% intact). Again, we could only characterize a few NSs, and therefore these conclusions can only be tentatively drawn.

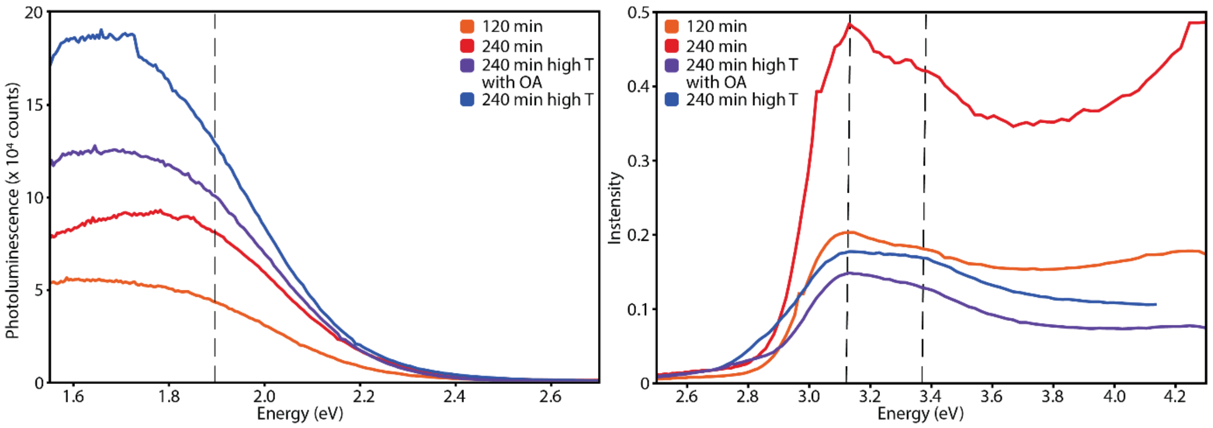

Figure S16. Photoluminescence emission and excitation measurements of completely exchanged CdS NSs. (a) We compare the emission of the CdS sheets after 120 and 240 minute experiments at 100°C and the 240 min high T experiment. When excited at 390 nm all samples show a broad sub-bandgap photoluminescence peak below 2.2 eV. With a prolonged reaction at higher temperature the sub-bandgap luminescence increases, potentially due to improved quality of the NSs upon annealing or as a result of the additional precursor. (b) The excitation spectra of the CdS NSs as monitored at 1.9 eV (650 nm) show the heavy-hole and light-hole exciton peaks at 3.1 eV and 3.35 eV respectively (indicated by the black dashed lines), while free carrier absorption sets on at 3.8 eV.

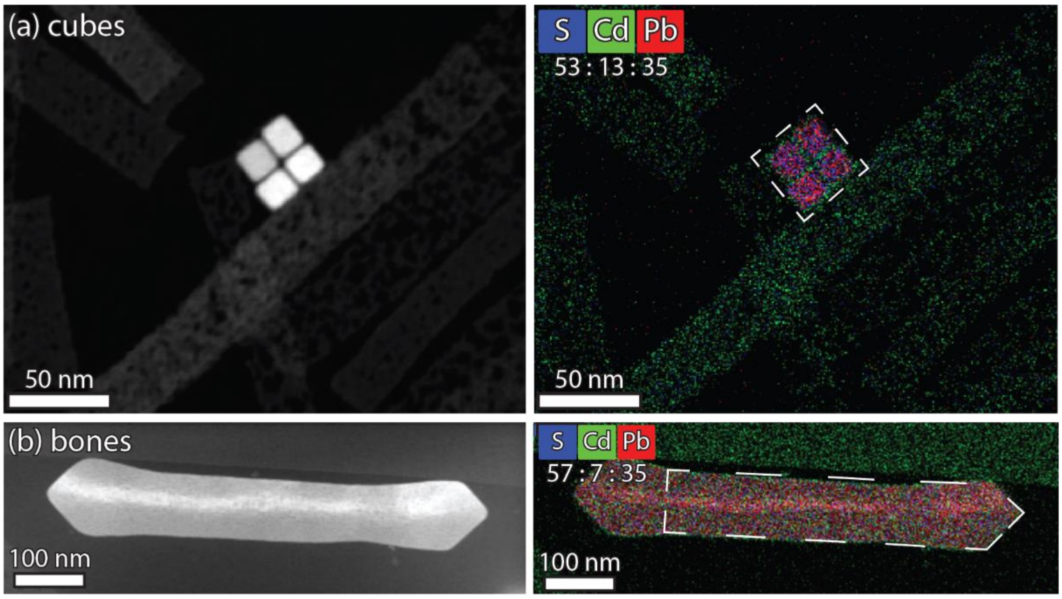

Figure S17. (a) As a by-product of the PbS NSs synthesis, rock salt PbS cubes can form with an average size of 16.7 nm. (b) Another by-product of the PbS NSs synthesis which can form are the "bones". These bones tend to form when the initial synthesis is prolonged at 165°C. After these by-products undergo a cation exchange procedure, they are surrounded by CdS NSs containing up to 43% cadmium. However, the cubes still have a PbS core with a thin shell of CdS (between 3 and 4 nm thick) while the bones show homogeneous incorporation in the analysed zone axis. Quantification of the areas indicated by the white boxes in the EDX maps shows that the cubes only contain 12.9% Cd, and the bone only contains 7.4 % Cd.

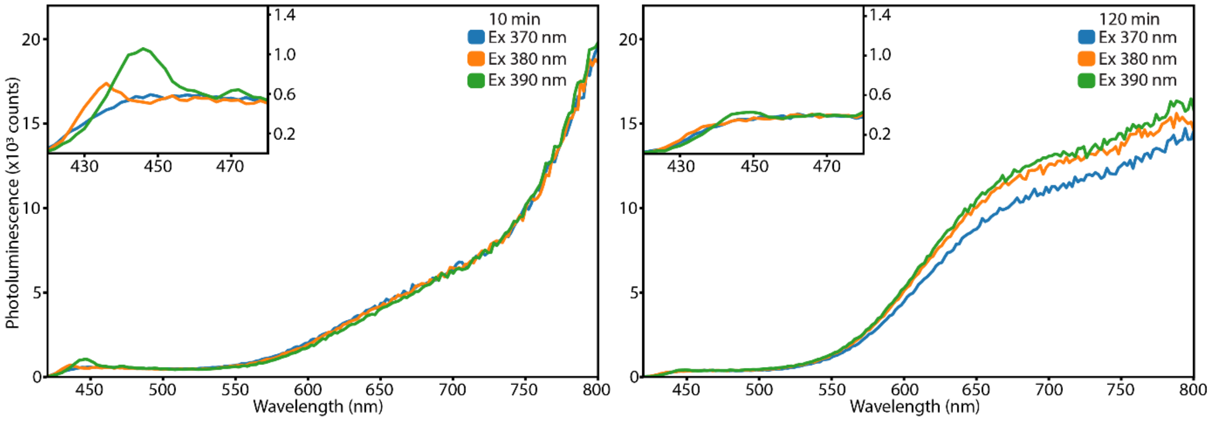

Figure S18. In the photoluminescence measurements a Raman scattering feature is observed around 446 nm. For both 10 minutes and 120 minutes of exchange, an inset is shown of the 420 to 480 nm area showing that this scattering depends upon the excitation wavelength and is less pronounced at longer reaction times.

REFERENCES

(1) van der Sluijs, M. M.; Salzmann, B. B. V.; Arenas Esteban, D.; Li, C.; Jannis, D.; Brafine, L. C.; Laning, T. D.; Reinders, J. W. C.; Hijmans, N. S. A.; Moes, J. R.; et al. Study of the Mechanism and Increasing Crystallinity in the Self-Templated Growth of Ultrathin PbS Nanosheets. *Chem. Mater.* **2023**, *35* (7), 2988-2998. DOI: 10.1021/acs.chemmater.3c00300.
(2) Moreels, I.; Lambert, K.; De Muynck, D.; Vanhaecke, F.; Poelman, D.; Martins, J. C.; Allan, G.; Hens, Z. Composition and Size-Dependent Extinction Coefficient of Colloidal PbSe Quantum Dots. *Chem. Mater.* **2007**, *19* (25), 6101-6106. DOI: 10.1021/cm071410q.
(3) De Roo, J.; Yazdani, N.; Drijvers, E.; Lauria, A.; Maes, J.; Owen, J. S.; Van Driessche, I.; Niederberger, M.; Wood, V.; Martins, J. C.; et al. Probing Solvent–Ligand Interactions in Colloidal Nanocrystals by the NMR Line Broadening. *Chem. Mater.* **2018**, *30* (15), 5485-5492. DOI: 10.1021/acs.chemmater.8b02523.
(4) Singh, S.; Leemans, J.; Zaccaria, F.; Infante, I.; Hens, Z. Ligand Adsorption Energy and the Postpurification Surface Chemistry of Colloidal Metal Chalcogenide Nanocrystals. *Chem. Mater.* **2021**, *33* (8), 2796-2803. DOI: 10.1021/acs.chemmater.0c04761.
(5) Klinger, M.; Jager, A. Crystallographic Tool Box (CrysTBox): Automated Tools for Transmission Electron Microscopists and Crystallographers. *J. Appl. Crystallogr.* **2015**, *48* (Pt 6), 2012-2018. DOI: 10.1107/S1600576715017252.
(6) Zagorodni, A. *Ion Exchange Materials Properties and Applications*; Elsevier, 2007.
